# Trends in hospital admissions for adrenal insufficiency in adolescents and young adults in the 21^st^ century

**DOI:** 10.3389/fendo.2022.986342

**Published:** 2022-09-20

**Authors:** Georgina L. Chrisp, Maria Quartararo, David J. Torpy, Henrik Falhammar, R. Louise Rushworth

**Affiliations:** ^1^ The University of Notre Dame, Australia, Darlinghurst, NSW, Australia; ^2^ Endocrine and Metabolic Unit, Royal Adelaide Hospital, Adelaide, SA, Australia; ^3^ University of Adelaide, Adelaide, SA, Australia; ^4^ Department of Endocrinology, Karolinska University Hospital, Stockholm, Sweden; ^5^ Department of Molecular Medicine and Surgery, Karolinska Institutet, Stockholm, Sweden

**Keywords:** adrenal, adrenal crisis, adrenal insufficiency, adolescence, emerging adults

## Abstract

**Background:**

Very little is known about the epidemiology of adrenal crises (AC) and adrenal insufficiency (AI) in adolescents and young adults.

**Methods:**

Data on all admissions to Australian hospitals between 2000/1 to 2019/20 for a principal diagnosis of AI (including AC) in 10-24 year olds were extracted from a national repository. Age and sex-specific rates and age-adjusted rates were compared.

**Findings:**

Over the study, there were 3386 admissions for a principal diagnosis of AI; 24.0% (n=812) were for an AC and 50·7% (n=1718) were for secondary AI. Age-adjusted AI admissions increased from 31·70/million in 2000/1 to 54·68/million in 2019/20 (p<0·0001). Age-adjusted AC admissions also increased, most notably in the second decade (from 5·80/million in 2010/11 to 15·75/million in 2019/20) (p<0·00001). Average AI and AC admission rates were comparable between the sexes, but rates increased significantly in females, especially in those aged 20 to 24 years, whose AC rate in 2019/20 (39·65/million) was significantly higher than the corresponding rate in 2000/1 (3·15/million) (p<0·00001). Average age-adjusted SAI admission rates were higher in males (23·92/million) than females (15·47/million) (p<0·00001). However, SAI admission rates increased only among females (from 11·81/million to 22·12/million in 2019/20), with an increase in 20-24 year old females in the second decade from 5·07/million in 2010 to 20·42/million (p<0·00001). Age adjusted admissions for congenital adrenal hyperplasia, primary AI (PAI) and drug-induced AI did not change significantly over the study.

**Interpretation:**

AC/AI admissions increased over the first two decades of this century in the emerging adult population, particularly among females who also experienced a marked increase in AC admission rates, most evident in the second decade. Although uncertain, possible explanations include: dose of glucocorticoid replacement; non-adherence to therapy; psychosocial factors; and difficulty in transition to adult services. Admissions for SAI also increased, while rates of PAI and CAH remained constant.

## Introduction

Adrenal insufficiency (AI) is a rare cause of morbidity and occasional mortality among adolescents and young adults (1–4). The estimated prevalence of AI in this age group is approximately 120/million, with aetiological factors that include congenital and autoimmune disorders, tumours, and traumatic brain injury (1–4). Congenital adrenal hyperplasia (CAH) is the commonest cause of primary AI (PAI) in children (incidence between 1/14000 and 1/18000) (4). Secondary AI (SAI) is less common in this age group, having an estimated prevalence of 25/million, with causes that include congenital anomalies, cerebral tumours and their treatment, and traumatic brain injuries (5). Glucocorticoid induced AI, on the other hand, is thought to be common, may require ongoing glucocorticoid replacement, and is often unrecognised.

All patients with AI are at risk of an adrenal crisis (AC), which is an acute episode of AI characterised by hypotension, electrolyte abnormalities (hyponatraemia and hyperkalaemia), alterations in consciousness, acute abdominal symptoms and hypoglycaemia (in children) (6, 7). Education of patients and their carers, especially with regard to domiciliary emergent glucocorticoid stress dosing (oral, intramuscular or subcutaneous, when necessary), is a key component in AC prevention and can be life-saving (6, 7). Other preventive measures include use of medical jewellery and carriage of a steroid dependency card (6 7). Despite efforts directed at education and prevention, ACs continue to occur at an estimated rate of 6 to 8 ACs/100 patient years in treated AI (8, 9).

AC episodes have been studied in infancy and early childhood, particularly in relation to AC incidence in CAH, and among adults (2, 8–11). However, the occurrence of ACs, and the epidemiology of AI more generally in adolescents and young adults, has not been a focus of research, despite the important personal and social challenges to the self-management of AI that feature during this period of transition into adulthood (12–14). The present study aims to address the paucity of information on AI/AC epidemiology among emerging adults by examining AC/AI hospitalisations in this age group in Australia between 2000/1 and 2019/2020.

## Methods

### Admission data

Information on all admissions to all Australian hospitals (public and private) is collected by each State or Territory health department. The Australian Institute of Health and Welfare (AIHW) stores these data, which are available for each Australian financial year (July 1 to June 30) in its Principal Diagnosis Datacubes (https://www.aihw.gov.au/reports/hospitals/principal-diagnosis-data-cubes/contents/data-cubes) according to year and principal diagnosis, coded using the International Statistical Classification of Diseases and Related Health Problems, Tenth Revision, Australian Modification Australian modification (ICD CM 11-AM) (15). Variables available in the datacubes and included in the dataset comprise: the principal diagnosis (main reason for the admission); age in five-year categories; sex; and financial year of admission (July 1 to June 30).

For this study, data were extracted from the AIHW for the years 2000/1 to 2019/20 for all admissions of adolescents and young adults aged 10 to 24 years for the following principal diagnoses: hypopituitarism (E23.0 and E23·1); post-procedural hypopituitarism (E89·3); congenital adrenal hyperplasia (E25); primary adrenal insufficiency (PAI) (E27·1); Addisonian (adrenal) crisis (E27·2); drug-induced adrenal insufficiency (E27·3) (excluding information on the agent causing AI); and “Other and unspecified adrenal insufficiency” (E27·4) (including secondary AI, tertiary AI and where the specific form of AI is not given).

For the years 2015/16 and 2016/17, data on the age and sex breakdown of CAH admissions were not published, so mean values of the remaining admissions for CAH for each age and sex group were substituted.

### Population data

Information on the age and sex structure of the Australian population is available from the Australian Bureau of Statistics (https://www.abs.gov.au/statistics/people/population/national-state-and-territory-population/latest-release#data-downloads-data-cubes). Data on the sex and age group (10-14, 15-19 and 20-24 years) of the population between 2001 and 2020 were downloaded to correspond with each year of the study.

### Statistical analysis

Downloaded data were entered into Microsoft Excel. Age and sex-specific admission rates were calculated for AC and AI subtypes (according to the ICD-10 CM AM code) for each year. Age-adjusted admission rates were calculated by using the population structure of the year 2000 as the base year.

Z-scores were calculated to assess the difference between admission rates in the first and last years of the study. Poisson regression models were constructed using R version 4·0·2 to assess the effect of secular trends in admission rates (presented as ‘trend’ with an accompanying p value) overall and for each age and sex group. ANOVA was used to assess the differences in mean admission rates between groups. Where results were only significant for certain sex and age groups, these are presented in isolation.

As the sample sizes for each year were large and multiple comparisons were conducted, a conservative p-value of p<0·001 was regarded as significant.

### Ethics

As all data used in these analyses were from publicly available datasets, no ethics clearance was required to conduct the study.

## Results

### Admissions for adrenal insufficiency

There were 3386 admissions for a principal diagnosis of AI or hypopituitarism over the study period, half (50·7%, n=1718) of which were for SAI; 24·0% (n=812) were for an AC; 16·6%, (n=561) were for primary AI; and 8·7% (n=295) were for CAH. Males comprised 52·4% (n=1774) of the sample; 39·5% (n=1339) were aged 10-14 years and fewer (27·4%, n=929) were aged 20-24 years.

**Figure 1:**
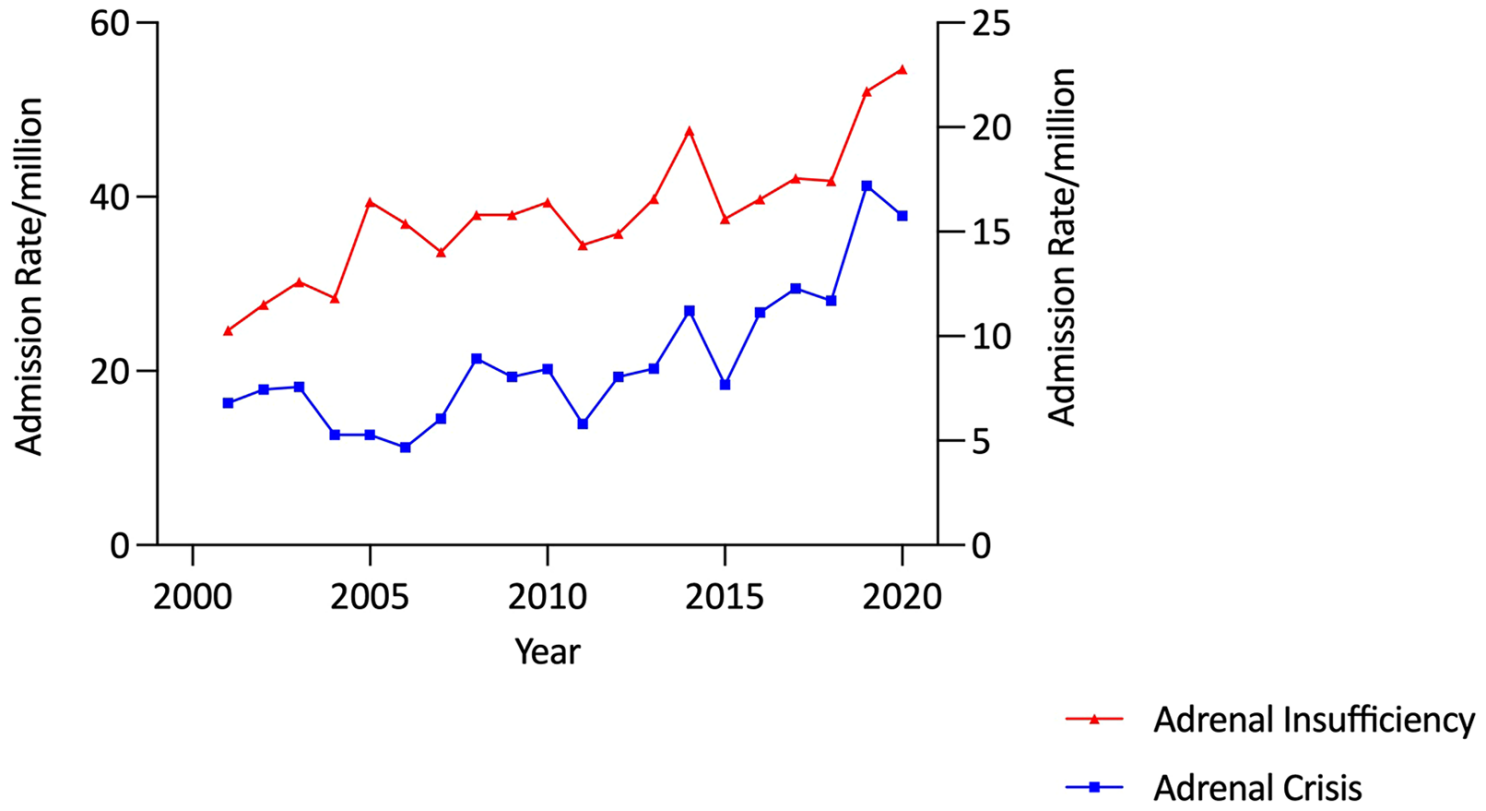
Adjusted adrenal insufficiency and adrenal crisis admission rates, 10-24 years, Australia, 2000/1 to 2019/20.

Annual AI admissions increased from 126 in 2000/1 to 263 in 2019/20, corresponding to a 72.5% increase in the age-adjusted total AI admission rate (from 31·70/million in 2000/1 to 54·68/million in 2019/20, p<0·00001) (**Figure 1**).

**Figure 2:**
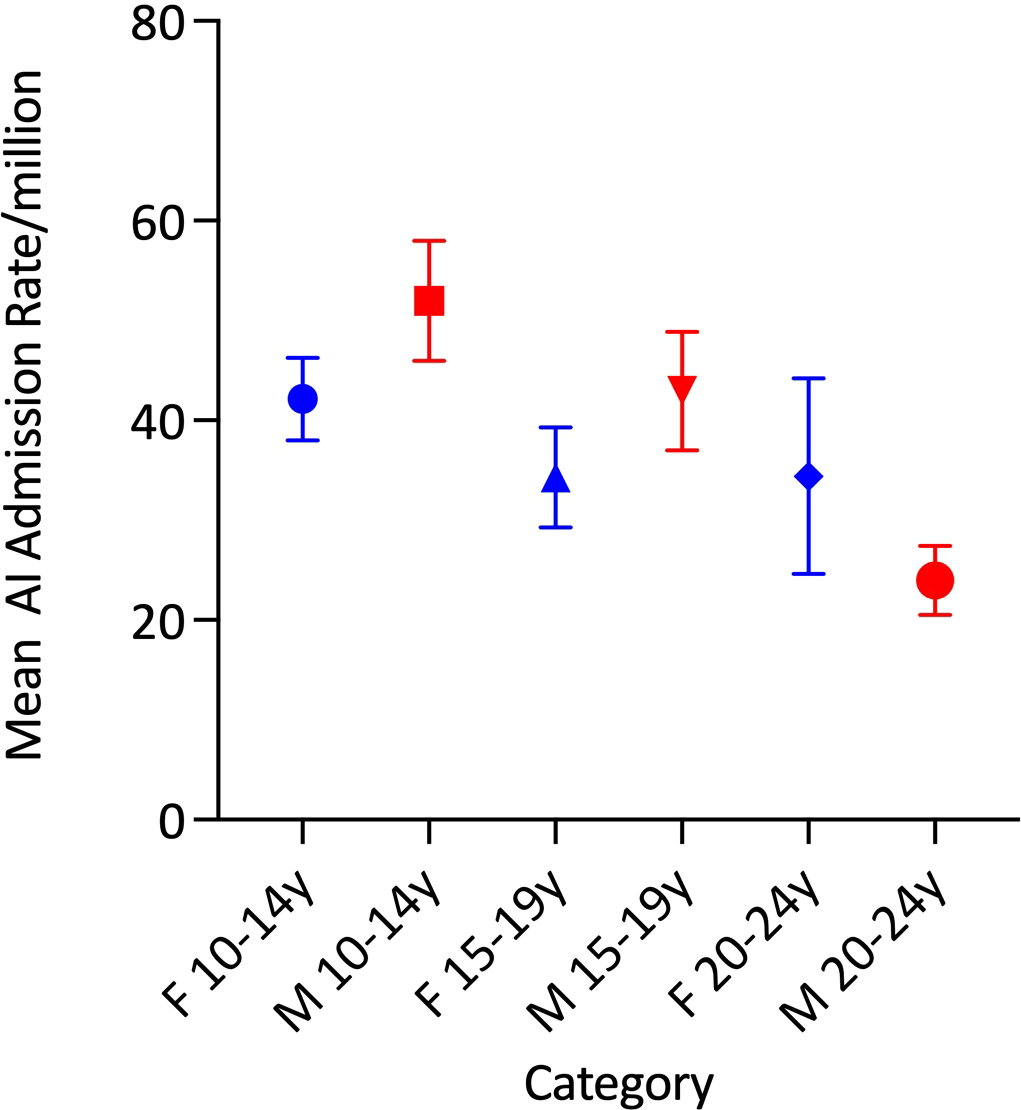
Mean total adrenal insufficiency admission rates by age group and sex.

Average age-adjusted admission rates were comparable between the sexes (males: 39·86/million and females: 36·98/million). Within the sexes, however, average age-specific admission rates did not vary significantly by age in females but differed according to age among males (p<0·00001) in whom the rate was highest in those aged 10-14 years and lowest among young adults (**Figure 2** and **Table 1**).

**Table 1:** Average admission rates by sex and age group and adrenal Insufficiency Subtype*.

		Male			Female			Total	
Age (years)	10-14	15-19	20-24	10-14	15-19	20-24	10-14	15-19	20-24
***Primary AI***									
PAI	4.1 (3.2, 5.1)	7.3 (5.5, 9.1)	6.6 (5.2, 8.0)	4.3 (3.1, 5.6)	7.9 (6.1, 9.7)	7.5 (5.4, 9.5)	4.2 (3.2, 5.3)	7.6 (5.8, 9.3)	7.0 (5.3, 8.7)
CAH	3.3 (2.0, 4.7)	2.3 (1.1, 3.6)	0.5 (-0.02, 1.0)	9.4 (7.5, 11.3)	3.8 (2.9, 4.6)	1.4 (0.8, 2.0)	6.3 (4.2, 8.5)	3.1 (2.0, 4.1)	0.9 (0.4, 1.5)
***Secondary AI***									
Hypopituitarism	25.9 (21.9, 29.9)	19.8 (15.1, 24.5)	6.6 (3.8, 9.4)	16.7 (14.3, 19.1)	7.5 (6.1, 8.9)	3.6 (1.8, 5.3)	21.3 (17.4, 25.1)	13.7 (9.3, 18.1)	5.1 (2.7, 7.4)
Other AI	10.3 (7.0, 13.7)	4.9 (2.9, 6.9)	2.0 (1.1, 2.9)	6.0 (4.2, 7.8)	5.1 (3.2, 7.1)	5.3 (3.1, 7.5)	8.2 (5.4, 10.9)	5.0 (3.1, 6.9)	3.6 (1.9, 5.4)
All SAI	37.1 (32.2, 42.0)	25.3 (20.2, 30.3)	8.9 (6.1, 11.8)	23.4 (20.1, 26.7)	13.4 (11.1, 15.6)	9.4 (6.1, 12.8)	30.3 (25.1, 35.4)	19.3 (14, 6, 24.0)	9.2 (6.2, 12.2)
**AC**	7.4 (5.5, 9.2)	8.0 (6.2, 9.8)	8.0 (6.2, 9.7)	5.0 (3.8, 6.2)	9.3 (6.6, 12.0)	16.1 (10.4, 21.7)	6.2 (4.6, 7.8)	8.7 (6.4, 10.9)	12.0 (7.6, 16.5)
All AI	51.9 (46.0, 57.8)	42.9 (37.0, 48.9)	24.0 (20.5, 27.4)	42.2 (38.0, 46.3)	34.3 (29.3, 39.3)	34.4 (24.6, 44.2)	47.0 (41.6, 52.5)	38.6 (33.0, 44.3)	29.2 (21.7, 36.7)

*Average admission rate/million (95% confidence intervals).

**Figure 3:**
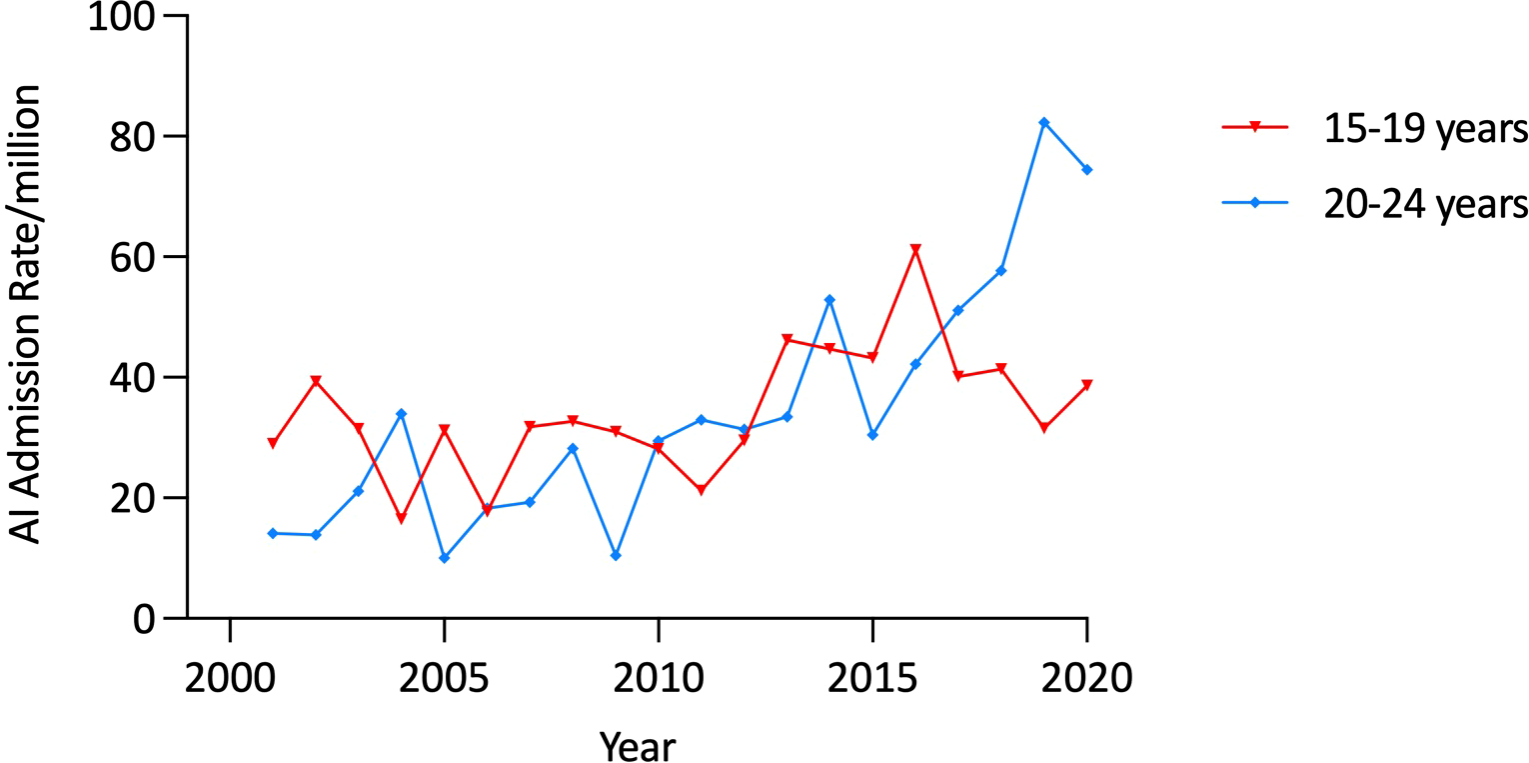
Age-specific admission rates for adrenal insufficiency in females aged 15 to 24 years, Australia, 2000/1 to 2019/20.

Admission rates did not change significantly over the study period in any age category among males or in females aged 10 to 14 years. However, in females aged 15-19 years, AI admission rates increased by 33·4% (from 28·97/million in 2000/1 to 38·64/million in 2019/20, trend, p<0·001) (**Table 2**). There was a more marked (426·1%), increase in the AI admission rate among 20 to 24 year old females (from 14·16/million in 2000/1 to 74·49/million in 2019/20, trend, p<0·00001), with the increase being most prominent in the period 2010-2020 (**Figure 3**).

**Table 2:** Admission rates for adrenaI insufficiency by sex and age group in 2000/1 & 2019/20*.

		Male			Female			Total		
		10-14	15-19	20-24	10-14	15-19	20-24	10-14	15-19	20-24
**2019/** **2020**	***Primary AI***									
	PAI	6.10	9.12	6.82	7.72	4.14	12.01	6.89	6.70	9.34
	CAH	0.00	1.30	2.27	3.86	5.52	2.40	1.88	3.35	2.34
	***Secondary AI***									
	Hypopituitarism	21.98	26.06	21.59	16.74	5.52	12.01	19.43	16.09	16.94
	Other AI	18.31	14.33	1.14	15.45	5.52	7.21	16.92	10.05	4.09
	All SAI	40.29	41.70	22.73	33.47	12.42	20.42	36.97	27.48	21.61
	**AC**	10.99	14.33	7.95	6.44	16.56	39.65	8.77	15.42	23.36
	All AI	57.38	66.46	39.77	51.50	38.64	74.49	54.52	52.95	56.65
**2000/1**		**Male**			**Female**			**Total**		
		**10-14**	**15-19**	**20-24**	**10-14**	**15-19**	**20-24**	**10-14**	**15-19**	**20-24**
	***Primary AI***									
	PAI	5.81	8.77	9.17	1.53	9.15	3.15	3.72	8.96	6.20
	CAH	8.72	1.46	0.00	6.10	3.05	1.57	7.44	2.24	0.78
	***Secondary AI***									
	Hypopituitarism	29.05	19.00	3.06	15.25	7.62	4.72	22.32	13.43	3.88
	Other AI	2.91	0.00	0.00	3.05	3.05	1.57	2.98	1.49	0.78
	All SAI	31.96	19.00	3.06	18.30	10.67	6.29	25.30	14.93	4.65
	**AC**	**2.91**	**10.23**	**16.81**	**1.53**	**6.10**	**3.15**	**2.23**	**8.21**	**10.08**
	All AI	49.39	39.46	29.03	27.45	28.97	14.16	38.69	34.33	21.70

*Rate/million.

### Admissions for adrenal crises

There were 812 AC admissions over the study period; 43·6% (n=354) of which were in males and nearly half (47·3%, n=384) were in patients aged 20-24 years. Admissions increased from 27 in 2000/1 to 77 in 2019/20, corresponding to a 132·0% rise in the age-adjusted AC admission rate from 6·79/million to 15·75/million (p<0·0001), with the increase being most marked in the second decade (**Table 2** and **Figure 1**).

**Figure 4:**
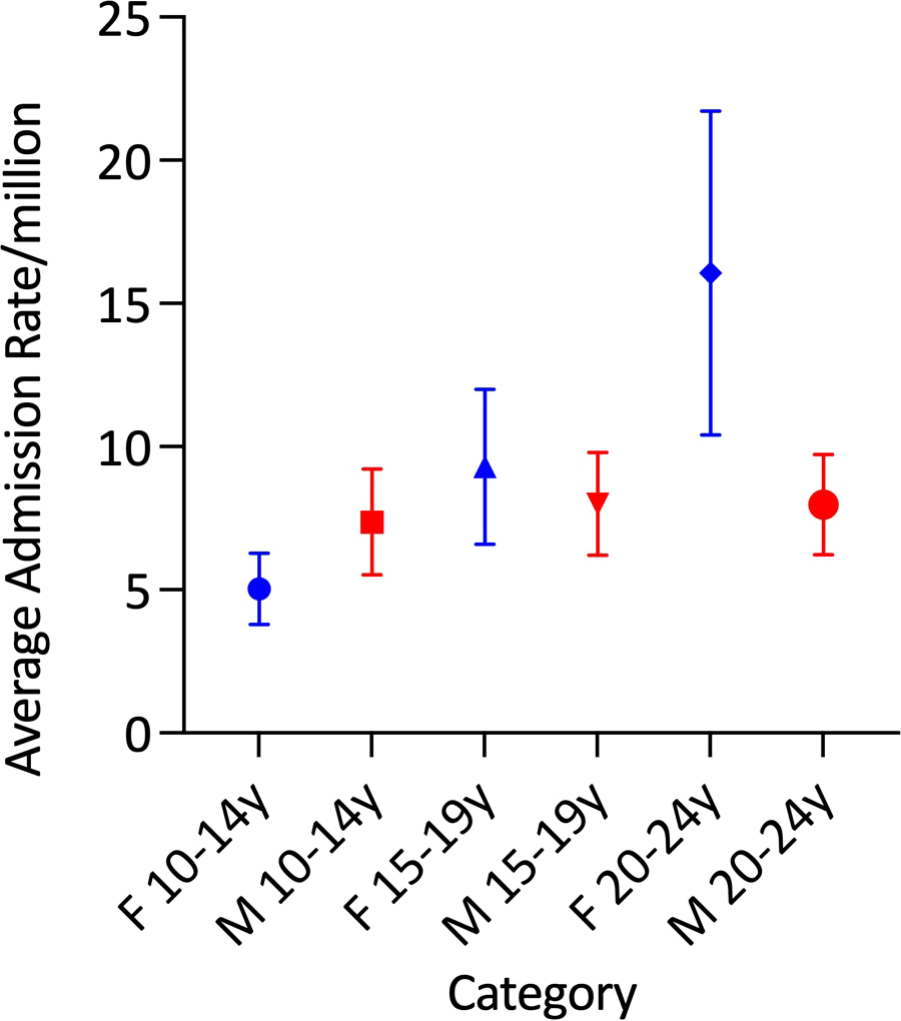
Mean adrenal crisis admission rates by age group and sex.

Average age-adjusted AC admission rates were comparable between the sexes (male: 7·79/million and female: 10·06/million). There was significant variation in the average age-specific AC admission rates in females (p<0·0001), but not in males (**Table 1** and **Figure 4**). Average AC admission rates among females were lowest in those aged 10-14 years (5·0/million) and highest in those aged 20-24 years (16·1/million) (**Figure 4**).

**Figure 5:**
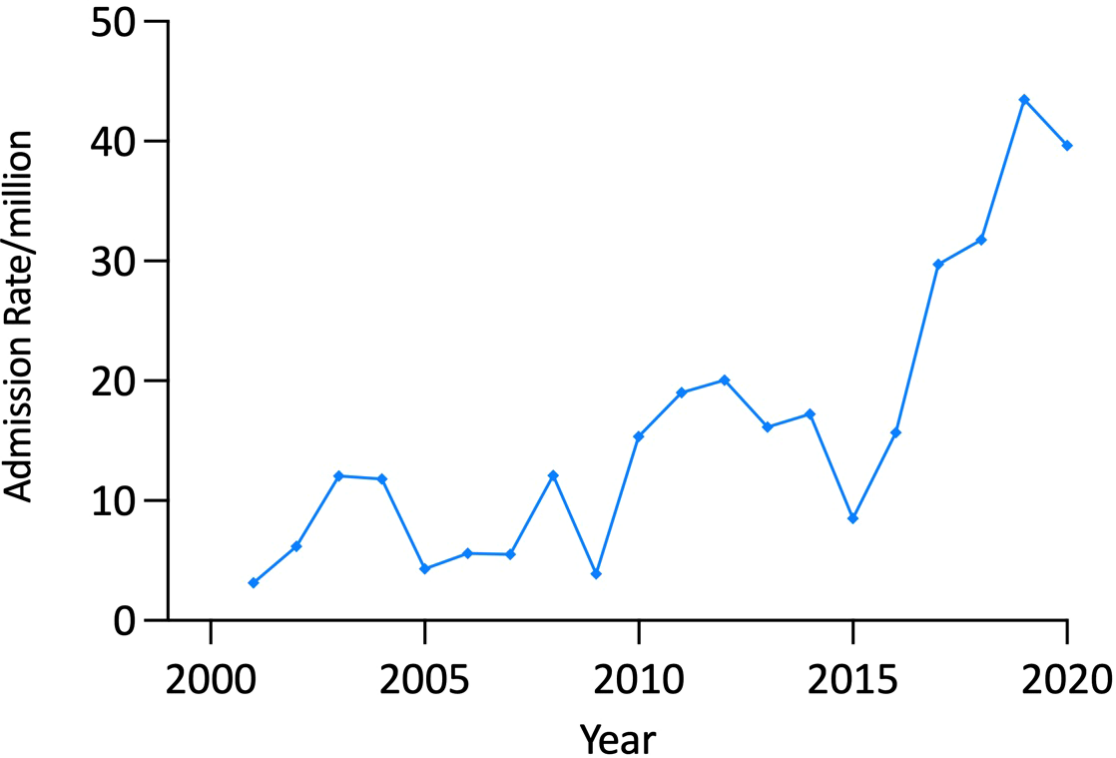
Adrenal crisis admission rate, females aged 20-24 years by year.

AC admission rates in males were steady (9.87/million in 2000/1 and 11.14/million in 2019/20, p=0.4) over the study period. In contrast, the female rate increased by 474·7%, from 3·60/million in 2000/1 to 20·69/million in 2019/20 (p<0·00001). Within the age-sex specific categories, a significant secular trend in AC admissions was identified only among females aged 20-24 years, in whom the AC admission rate increased substantially. In this group, admission rates rose from 3·15/million in 2000/1 to 39·65 in 2019/20, which included a 152·9% increase in the last 5 years of the study (from 15·68/million in 2015/16 to 39·65 in 2019/20) (trend p<0·00001) (**Figure 5**).

### Admissions for secondary adrenal insufficiency

**Figure 6:**
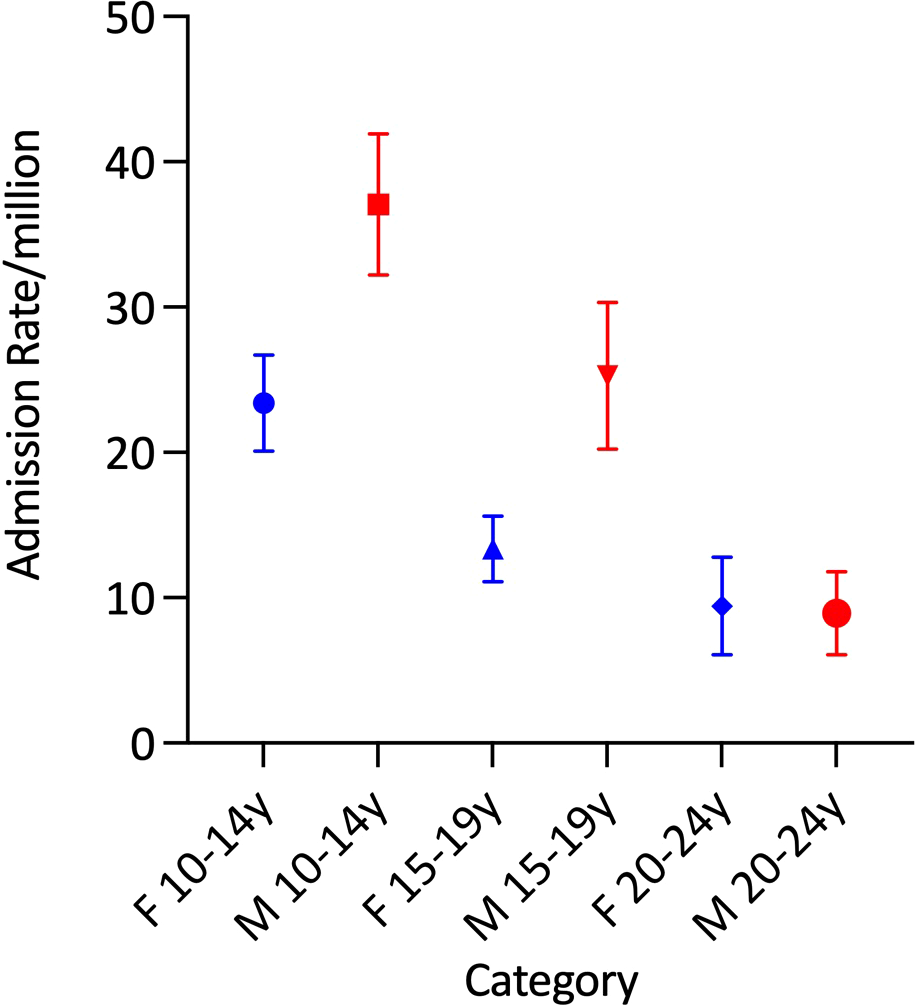
Mean secondary adrenal insufficiency admission rates by age group and sex.

There were 1718 admissions for the combined SAI group (hypopituitarism, “other AI” and drug-induced AI). Admissions rose from 60 in 2000/1 to 137 in 2019/20, corresponding to a 90·6% increase in the age-adjusted admission rate from 15·10/million in 2000/1 to 28·78/million in 2019/20 (trend, p<0·00001). Males comprised 61·5% (n=1056) of these admissions, corresponding to an average adjusted SAI admission rate of 23·92/million which was higher than that in females (15·47/million) (p<0·00001). There was significant variation in the average SAI admission rate in both sexes according to age group (both p<0·00001), with the average rate decreasing with age (**Table 1** and **Figure 6**).

**Figure 7:**
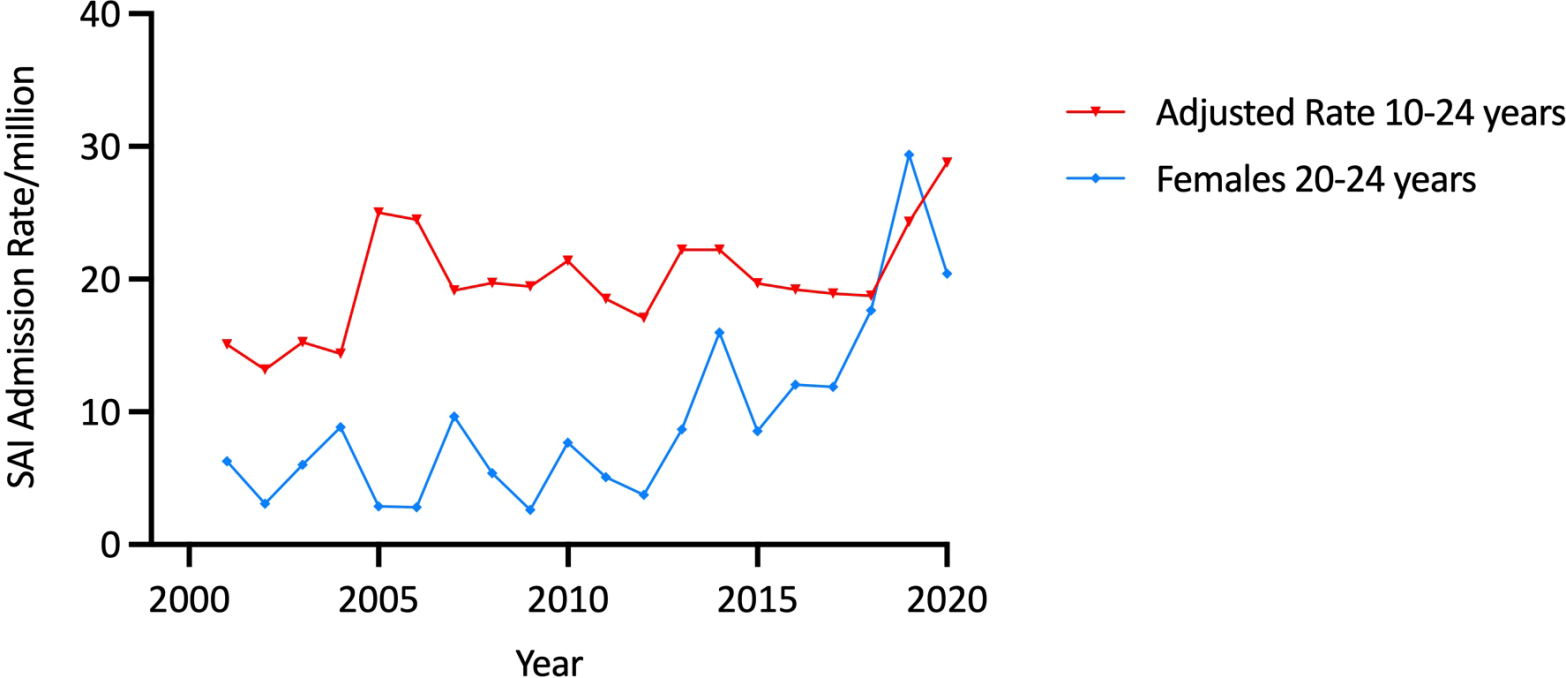
Age-adjusted 10-24 years and female 20-24 year admission rates for secondary adrenal insufficiency by year, Australia, 2000/1 to 2019/20.

Significant secular trend increases over the study period were observed in females, whose SAI admission rates increased by 87·3% (from 11·81/million to 22·12/million, trend p<0·00001) but similar changes were not evident among males. Within age-sex-specific categories, significant secular trends (p<0·00001) were only identified in young adult females, whose admission rates increased by 302·8% over the second decade (from 5·07/million in 2010 to 20·42/million in 2019/20) after being stable in the first decade (**Table 2** and **Figure 7**).

### Admissions for “other” adrenal insufficiency

**Figure 8:**
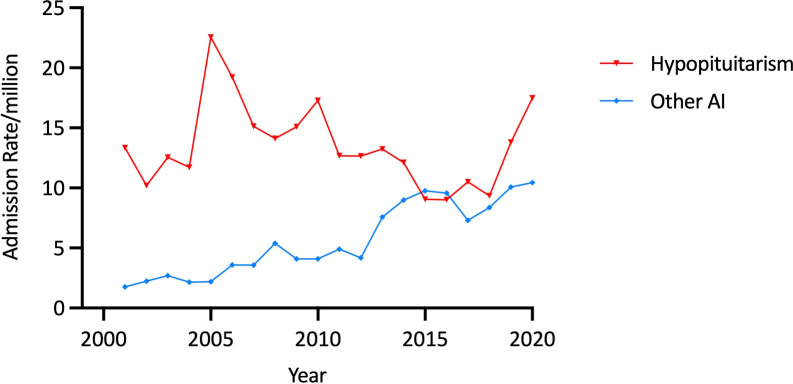
Age-adjusted admission rates for “Other AI” and hypopituitarism, 10-24 years, Australia, 2000/1 to 2019/20.

Of the 502 admissions for a principal diagnosis of “Other AI”, 51·8% (n=260) were in males. Overall, the age-adjusted “Other AI” admission rate rose by 493·2%, from 1·76 in 2000/1 to 10·44/million in 2019/20 (trend p<0·00001) (**Figure 8**). The average age-adjusted male admission rate (5·81/million) was comparable to the female rate of (5·48/million). Among males, average age-specific admission rates decreased with age (p<0·00001), with the highest rate being among males aged 10-14 years (10·33/million) but there was no difference in mean rates according to age among females.

Age-adjusted admission rates increased in both males (from 0·99/million to 9·85/million in 2000/1 to 2019/20) and females (2·57/million in 2000/1 to 10·37/million in 2019/20) (both p<0·00001). Within the age-sex specific categories, admission rates for “Other AI” increased in the younger two age groups in males (both p<0·00001) and in the 15 to 19 year and 20 to 24 year age group in females (both p<0·00001) (**Table 2**).

### Admissions for hypopituitarism

There were 1160 admissions for hypopituitarism over the study period, two thirds (66·3%, n=769) of which were in males; 52·1% (n=604) were in aged 10-14 years, and 13·8% (n=160) were in the oldest age group. Age-adjusted admissions for hypopituitarism increased from 13·34/million in 2000/1 to 22·56/million in 2004/5 and then decreased to 17·49/million in 2019/20 (**Figure 8**).

Average age-adjusted rates were higher in males than females (17·60/million vs 9·32/million, p<0·0001). In both sexes, average admission rates decreased with age (both p<0·00001) (**Table 1**). No significant secular changes in hypopituitarism admission rates were identified on regression modelling.

### Admissions for drug-induced adrenal insufficiency

Of the few (n=56) admissions with a principal diagnosis of drug-induced AI, 48·2% (n=27) were in males and few (n=18) were recorded in the 15 to 19 year or the 20 to 24 year (n=15) groups. Age-adjusted admission rates increased from 0·0/million in 2000/1 to 0·85/million in 2019/20 (p=0.3). There were no significant secular trends identified.

### Admissions for primary adrenal insufficiency

Of the 561 admissions for a principal diagnosis of PAI, 51·3% (n=288) were in females; 39·0% (n=219) were in patients aged 15-19 years and 39·6% (n=222) were for patients aged 20-24 years. Age-adjusted PAI admission rates were constant over the study period; 6·29/million in 2000/1 and 7·62/million in 2019/20.

Average age-adjusted admission rates were 5·99/million for males and 6·55/million in females, and there were no significant differences in the age-specific admission rates in either sex (**Table 1**). No significant secular trends were identified in age-sex specific PAI admission rates over the time period.

### Admissions for congenital adrenal hyperplasia

Of the 295 admissions for a principal diagnosis of CAH, 69·2% (n=204) were in females. Age-adjusted admission rates for CAH remained stable over the study period (3·52/million in 2000/1 and 2·52/million in 2019/20). Average age-adjusted admission rates were 4·89/million in females and 2·07/million in males. Among females, average age-specific admission rates diminished with increasing age (p<0·00001), but rates did not change with age among males (**Table 1**). No significant secular trends in the age-sex specific categories were identified.

## Discussion

This study is the first to analyse trends in hospital admissions for AI/AC specifically in adolescents and young adults, a population about which little is known regarding the epidemiology of AI. It showed that, between 2000/1 and 2019/20, admissions for both AI and AC increased but that there was a greater increase in AC relative to AI admissions. The secular changes in AI/AC admissions were most evident in the second decade, especially among women aged 20-24 years, in whom AC admission rates increased from 3·15/million in 2000/1 to 39·65/million in 2019/2020. Although unknown, possible reasons for this increase include: psychosocial factors; difficulties with transition to adult health services; non-adherence to therapy or lower dosing of glucocorticoid replacement. Admission rates for all forms of SAI also increased, particularly in females. Over the same period, there was a substantial rise in “Other AI” admissions, which was partially offset by a relative decline in admissions for hypopituitarism which occurred in both sexes and across the age-spectrum. This suggests a change in diagnostic classification of patients, possibly to reflect more accurately the reason for hospitalisation in patients with SAI and at least one other pituitary hormone deficit. In contrast, admissions for PAI, CAH and drug-induced AI remained constant, with the latter two diagnoses being rare in this population.

The reason for the increase in AI and AC admissions is uncertain, although the comparatively greater rise in AC relative to AI admissions in the second decade suggests an increase in severity of presentations. This may be attributable, at least in part, to the uptake of recommendations advocating use of lower doses of short acting glucocorticoid replacement therapy (hydrocortisone or cortisone acetate) which may expose patients to periods of hypocortisolaemia, potentially predisposing them to an AC (9, 16, 17). Alternatively, diagnostic classifications of AI/AC may have evolved, leading to an increased use of “AC” when previously the more general “AI” diagnosis may have been used (18). However, the variations in AC rate increases identified between the age and sex groups in this study suggest that a generalised trend to diagnose patients who may be symptomatic but not necessarily severely ill with an AC rather than “AI” did not occur. Increased AC rates have also been observed among older adults in the same population, suggesting that factors underpinning the observed change affect a wide range of patients and are persistent (16).

Although average AC admission rates were comparable between the sexes, and admissions increased in both males and females, the increase among females was considerably larger than that in males, especially in the 20-24 year age group, despite relative parity in the underlying epidemiology of AI and its causes between the sexes. The reasons for this gender disparity are unknown. While physiological changes during puberty often require recalibration of glucocorticoid replacement doses, this would not be a prominent issue in young adults. One possible contributory factor may be the modern approach to glucocorticoid replacement, mentioned above, which is recommended for all patients but, possibly due to greater concern about steroid-related weight gain, may be used by some females at a lower dose/BSA than males. Eating disorders also tend to arise in this age group, are more common in females, and have been shown to increase the incidence of diabetic ketoacidosis (DKA) in young women with type 1 diabetes mellitus (T1DM) (19). By extension, eating disorders among young adult females with AI may be associated with patient-initiated dose omission or reduction in the prescribed daily dose of glucocorticoid replacement therapy, which would increase AC risk. Patients in this age group may also present with an AC in the context of previously undiagnosed AI, often after several attendances for health care with typical symptoms but without appropriate treatment (20).

Variation in AC predisposition has been reported in cohort studies in which some patients had no AC events while others had more than one episode (8, 9). Recurrent admissions for individuals could not be identified in this study but it is likely that some patients contributed more than one AI/AC admission. In the more common endocrine emergency, DKA, clinical experience, together with evidence from a recent meta-analysis, suggests that psychosocial factors appear to account for more frequent hospital presentations in some patients (21–23). In Australia, DKA incidence is highest among 15-19 year old females and, in the young adult age group, there is a female predominance at a ratio of 1·4:1 to males (22). DKA is also more common in lower socioeconomic groups (21, 22). Overall, about 40% of cases of DKA are thought to be due to non-adherence with the treatment regimen (22). Given that AI and T1DM both require daily commitment to self-management and changes to protocols during intercurrent illness, it is likely that similar psychosocial factors to those identified in T1DM patients would influence self-management in emerging adults with AI, and that this may be reflected in AC incidence, including the high rate of AC events in women aged 20-24 years (2). This phenomenon needs further exploration, particularly in light of the secular trends observed in this study.

Admissions for SAI were more common in males, which is consistent with evidence from previous studies, but females experienced a greater increase in SAI admission rates, to reach near equivalence in the last year of the study (24). Males in this age group are considered to have a higher prevalence of SAI due to increased rates of traumatic brain injury and cerebral tumours (and their treatment) (6, 24, 25). In this study, the proportionate increase in SAI among females exceeded the 14-fold increase in AC rates in young adult women, a pattern of occurrence that requires further investigation, both with regard to the aetiology of SAI and to the identification of the underlying AC precipitants. While pituitary adenomas increase in prominence as a cause of SAI in adulthood, and are typically identified earlier in females due to menstrual disturbances, this does not explain the increase in SAI admissions or the disparity in rates of change between the sexes found in this analysis. Contributory factors may be increased cerebral imaging uncovering asymptomatic pituitary tumours in young women resulting in treatment-related SAI, and an unrelated but possible increase in autoimmune hypophysitis (26, 27).

Studies have shown that adolescents and young adults with chronic health conditions, such as AI, face a range of challenges and have unique vulnerabilities as they progress from reliance on specialist paediatric care and parental oversight to effective self-management (14). In Australia, this typically occurs after 18 years of age, when young people are required to move from the paediatric environment to the less well-defined setting of adult healthcare services. Accessing developmentally appropriate support for these patients may be difficult, particularly in non-urban areas, and financial burdens may lead to lower levels of engagement with healthcare services and lower levels of use of appropriate management and AC preventative strategies in this age group (28). It is likely that these vulnerabilities vary between individuals, and that other factors, such as use of recreational drugs and alcohol, would also affect treatment adherence and increase AC risk (18).

This analysis found that admissions for a principal diagnosis of CAH were rare, despite this being the most common underlying cause of AI in young people. This may be indicative of the phenomenon of “missing” CAH patients that has been reported following discharge of individuals with CAH from specialist paediatric care (29). It is possible that teenage and young adult patients with CAH were admitted with an AC or were included in the “Other AI” diagnostic category when admitted for treatment of symptomatic AI. Alternatively, their AI diagnosis may be seen as a secondary problem relative to other reasons for admission, such as management of fertility and genitourinary issues. Admissions for a principal diagnosis of “drug-induced AI” were also very uncommon, despite drug-related iatrogenic AI being considered a common, often undiagnosed, and potentially increasing cause of AI in populations (30). It is possible that, in this dataset of hospital admissions, some patients coded as having an AC or another subtype of AI may have had drug-induced AI, thereby leading to an underestimate of the true rate of admissions for this category of AI. In addition, patients with comorbid drug-induced AI who were admitted for another related principal diagnosis, such as asthma, would not have been identified in this analysis.

The data used in this study are from a large, nationwide, population-based database of all admissions for a principal diagnosis of AI/AC spanning 20 years. As the data were for the principal diagnosis of each admission only, the rates of admission found in this study would be an underestimate of the true AI-related admission rate in this population. Whether a sick patient was diagnosed as having an AC or the less severe, AI (or its subtypes), was dependant on the diagnostic criteria used by the treating clinician. As there was only one diagnosis per patient, when an AC was nominated as the principal diagnosis, neither the underlying subtype of AI nor the precipitant of the AC could be determined. Further, these data were not matched for individual patients and, therefore, the influence of a small group of patients having repeat admissions could not be addressed. Age-adjustment was used to control for the effect of changing population demographics and a conservative level of statistical significance was chosen to address the effects of large sample sizes and multiple comparisons.

In conclusion, this is the first study to comprehensively examine national rates of hospital admissions for AI/AC in adolescents and young adults over the most recent twenty years. Increases in AI/AC admissions in these patients, particularly among females aged 20-24 years, are of concern, have not been reported previously, and are unexplained. Possible reasons include psychosocial factors, problems with transition to adult services; non-adherence or patient-initiated changes to medication; or type and dose of glucocorticoid replacement therapy. Given the patterns observed and knowledge of behavioural and social influences that increase the vulnerabilities of patients in this age group, the causes of the increase are likely to be multifactorial and complex and require further investigation.

## Data availability statement

Publicly available datasets were analyzed in this study. This data can be found here: https://www.aihw.gov.au/reports/hospitals/principal-diagnosis-data-cubes/contents/data-cubes.

## Ethics statement

Ethical review and approval was not required for the study on human participants in accordance with the local legislation and institutional requirements. Written informed consent from the participants’ legal guardian/next of kin was not required to participate in this study in accordance with the national legislation and the institutional requirements.

## Author contributions

GC: downloading data, age standardisation and analysis and preparation of results and manuscript. MQ: interpretation of analysis and results and manuscript preparation. DT: planning, interpretation of results and manuscript preparation. HF: interpretation of results and manuscript preparation. RR: analysis and preparation of results and manuscript. All authors contributed to the article and approved the submitted version.

## Conflict of interest

The authors declare that the research was conducted in the absence of any commercial or financial relationships that could be construed as a potential conflict of interest.

## Publisher’s note

All claims expressed in this article are solely those of the authors and do not necessarily represent those of their affiliated organizations, or those of the publisher, the editors and the reviewers. Any product that may be evaluated in this article, or claim that may be made by its manufacturer, is not guaranteed or endorsed by the publisher.
